# Molecular Cloning and Characterization of WRKY12, A Pathogen Induced WRKY Transcription Factor from *Akebia trifoliata*

**DOI:** 10.3390/genes14051015

**Published:** 2023-04-29

**Authors:** Feng Wen, Xiaozhu Wu, Lishen Zhang, Jiantao Xiao, Tongjian Li, Mingliang Jia

**Affiliations:** 1 Anhui Chuju Planting and Deep Processing Engineering Research Center, School of Biological Science and Food Engineering, Chuzhou University, Chuzhou 239000, China; 2 State Key Laboratory of Ecological Pest Control for Fujian and Taiwan Crops, Key Laboratory of Biopesticides and Chemical Biology, Ministry of Education, College of Plant Protection, Fujian Agriculture and Forestry University, Fuzhou 350002, China; 3 School of Pharmacy and Life Science, Jiujiang University, Jiujiang 332000, China

**Keywords:** WRKY12, *Akebia trifoliata*, disease resistant, transcription factor

## Abstract

WRKY transcription factors (TFs), which are plant-specific TFs, play significant roles in plant defense. Here, a pathogen-induced WRKY gene, named *AktWRKY12*, which was the homologous gene of *AtWRKY12*, was isolated from *Akebia trifoliata*. The *AktWRKY12* gene has a total length of 645 nucleotides and an open reading frame (ORF) encoding 214 amino acid polypeptides. The characterizations of AktWRKY12 were subsequently performed with the ExPASy online tool Compute pI/Mw, PSIPRED and SWISS-MODEL softwares. The AktWRKY12 could be classified as a member of WRKY group II-c TFs based on sequence alignment and phylogenetic analysis. The results of tissue-specific expression analysis revealed that the *AktWRKY12* gene was expressed in all the tested tissues, and the highest expression level was detected in *A. trifoliata* leaves. Subcellular localization analysis showed that AktWRKY12 was a nuclear protein. Results showed that the expression level of *AktWRKY12* significantly increased in *A. trifoliata* leaves with pathogen infection. Furthermore, heterologous over-expression of *AktWRKY12* in tobacco resulted in suppressed expression of lignin synthesis key enzyme genes. Based on our results, we speculate that AktWRKY12 might play a negative role in *A. trifoliata* responding to biotic stress by regulating the expression of lignin synthesis key enzyme genes during pathogen infection.

## 1. Introduction

All plant species are continually under attack from a vast range of pathogens, including viruses, bacteria, oomycetes, fungi and so on [1,2]. For example, rice crops are affected by almost 70 kinds of pathogens, including 8 viruses, 5 bacteria and about 50 fungi [3]. During millions of years of co-evolution, the relationship between plant and pathogen populations has become more complicated [4,5,6]. Pathogens can damage commercial crops and reduce production. For example, in previous studies, the production and quality of medicinal materials decreased rapidly when medicinal plants were subjected to disease [7,8,9]. Thus, protection of commercial crops, especially medicinal plants, against plant diseases is crucial for minimizing the losses and increasing total production and quality. In the past few decades, due to the application of highly toxic pesticides, large doses of pesticides or ignoring the safety interval of pesticide application and other problems, there may be risks to people, animals and the environment [10]. Deploying resistance genes in medicinal plants is the best way to eliminate plant diseases and reduce environmental damage by avoiding the application of agrochemicals.

Plant resistances were activated in plant cells upon pathogen infection, dependent on an intricate regulatory network of signaling pathways involving innate immunity and a class of resistance genes [11,12,13]. Transcription factors are important components of these disease response and resistant signaling pathways, in which WRKY transcription factors (TFs), which are plant-specific TFs, play significant roles in plant defense [14,15]. Members of this family contain at least one conserved DNA-binding domain, which consists of 60 amino acids, including a highly conserved WRKYGQK heptapeptide sequence at the N-terminal and a C_2_H_2_- or C_2_HC-type of zinc finger motif in its C-terminal. The WRKY domain is responsible for binding to a *cis*-element (W box), which is abundant in the promoter region of defense-related genes [16,17]. In general, group I WRKY proteins contain two WRKY domains, and group II WRKY proteins only contain one WRKY domain followed by a C_2_H_2_-type of zinc finger motif, while the rest of the members are grouped in group III. Numerous reports have demonstrated that WRKY TFs can help plants enhance their resistance to pathogens through up-regulating expression levels of the *PATHOGEN-RELATED* (*PR*) gene and inducing phytoalexin accumulation [18,19,20]. For example, Salicylic acid (SA)-induced WRKY TFs act upstream of non-expressors of pathogenesis-related gene 1 (NPR1) and mediate its expression as a positive regulator during the activation of plant defense responses [21]. In Arabidopsis, AtWRKY33 regulates the expression of camalexin biosynthetic genes as the downstream of (mitogen-activated protein kinase) MAPK cascade, thereby driving metabolic flow to synthesize camalexin and improve the seedling disease resistance. A novel tobacco WRKY transcription factor, NtWRKY12, is required to induce PR-1a expression via salicylic acid and bacterial elicitors [22,23]. In addition, many reports demonstrated that WRKY factors acted as negative regulators in plant defense responses [24,25]. It has been reported that *Gossypium hirsutum WRKY25* over-expression could enhance the susceptibility of plant response to fungal pathogens through reducing the transcription levels of SA or Ethylene (ET) signal-related genes, indicating that the reduction in pathogen resistance caused by GhWRKY25 might be related to the cross-talk of the SA and jasmonic acid (JA)/ET signaling pathways [26]. Journot-Catalino et al. [25] reported that two of Arabidopsis WRKY IId’s subfamily members, WRKY11 and WRKY17, acted as negative regulators of basal resistance to *Pseudomonas syringae* pv tomato. Further, two orthologs of AtWRKY12, *Medicago truncatula* sugar transporter (MtSTP) and PtrWRKY19 have been proven to negatively regulate the formation of secondary walls in pith cells, implying that AtWRKY12 might negatively regulate lignin biosynthesis [27,28].

*A. trifoliata* (Thunb.) Koidz., a member of *Lardizabalaceae* family, is mainly distributed in China, South Korea, and Japan [29]. It is a famous traditional Chinese medicinal plant because of its medicinal value. Its air-dried stems and fruits have been used as anti-inflammatory, anti-tumor and diuretic agents for a long time in China [30]. Moreover, the fresh fruit of *A. trifoliata* as a delicious fruit has long been eaten by the local people [31]. In recent years, with the excessive exploitation of natural resources and grave pollution of the environment, the wild resources of *A. trifoliata* are on the verge of exhaustion. Because of its value as both medicine and food, *A. trifoliata* has become an artificial planting commercial crop in the middle and lower reaches of the Yangtze River basin in China. However, the yield and quality of the medicinal materials and fruit of *A. trifoliata* have rapidly decreased because of the biotic stresses. Thus, protecting *A. trifoliata* from pathogens is a crucial way to increase total production and quality. In the fields, orchardman usually use agrochemicals against plant pathogens, which might decline the quality of fruit and medicinal materials. Therefore, it is important to improve the disease resistance and immunity of *A. trifoliata* seedlings. Here, we isolated a pathogen-induced WRKY transcription factor, AktWRKY12, from *A. trifoliata* leaves infected with *Colletotrichum acutatum*. A variety of bioinformatics methods and tools were used to analyze the sequence characteristics and phylogenetic trees of WRKYs from different plants. The expression patterns of *AktWRKY12* in different organs were further determined. Furthermore, over-expression of *AktWRKY12* in tobacco resulted in suppressed expression of key enzyme genes in lignin synthesis. Our data will provide useful information for further study of the role of WRKYs in the disease resistance signaling pathway.

## 2. Materials and Methods

### 2.1. Plant Materials and Growth Conditions

*A. trifoliata* seedlings were planted in Mutong yard in Jiujiang University, Jiangxi, China. Seeds of tobacco (*Nicotiana benthamiana*) were germinated on a medium containing 1/2 Murashige and Skoog’s (MS) medium in the growth chambers at 25 °C. The Illumination period of seedlings was 16 h light/8 h dark cycle. All samples were frozen in liquid nitrogen and refrigerated at −80 °C if RNA extraction and subsequent analysis were not performed immediately. All experiments were performed at least twice, and each of which had three biological replicates and presented the representative results of one experiment.

### 2.2. RNA Isolation and Amplification of AktWRKY12

Total RNA was isolated from plant samples using Trizol (Invitrogen, Carlsbad, CA, USA) method according to the manufacturer’s protocol. Genomic DNA contamination was removed using RNase-free DNaseI (TaKaRa, Dalian, China). The first strand cDNA was synthesized using PrimeScript^TM^ 1st Strand cDNA Synthesis Kit according to the manufacturer’s specification (TaKaRa, Dalian, China). cDNA from disease resistance variety of *A. trifoliata* was used as the template to amplify *AktWRKY12* using PrimeSTAR HS DNA Polymerase (TaKaRa, Dalian, China) with primer pairs (FP: 5′-ATGGAAGGAGATCGAGAAG-3′ and RP: 5′-TTAAAATGAGCTGAAGCATTC-3′). The reaction parameters were as follows: denaturation at 94 °C, annealing at 54 °C and extension at 72 °C, 30 cycles. The product was purified and cloned into pEASY-Blunt Cloning Kit (Transgen, Beijing, China), transformed into *Escherichia coli* strain DH5α and sequenced using Sangon Biotech (Shanghai, China).

### 2.3. Bioinformatics Analysis

The DNAMAN version 7 and NCBI CDD tools (http://www.ncbi.nlm.nih.gov/, accessed on 10 April 2022) were used to translate the DNA sequence and analyze the conserved domain of WRKY proteins, respectively. The ExPASy online tool Compute pI/Mw (http://web.expasy.org/compute_pi/, accessed on 11 April 2022) was used to calculate the molecular weight (MW) and theoretical isoelectric point (pI) of deduced AktWRKY12 protein. The online subcellular location predictor CELLO (cello.life.nctu.edu.tw, accessed on 11 April 2022) was used to predict the subcellular localization with the default parameter. Gene ontology prediction results were performed using the PredictProtein online server (http://www.predictprotein.org, accessed on 12 April 2022). The secondary structure and 3D molecular modeling of AktWRKY12 protein was predicted through PSIPRED analysis (http://bioinf.cs.ucl.ac.uk/psipred/, accessed 12 April 2022) and SWISS-MODEL (http://swissmodel.expasy.org/, accessed on 12 April 2022) with the default parameters, respectively. The multiple alignment analyses of the WRKYs sequences via ClustalW and were displayed in GeneDoc software. Neighbor-joining (NJ) and Maximum likelihood (ML) trees were performed using the MEGA-X tools, based on the alignment of WRKY12s protein sequences in different species (parameters selected: p-distance, pairwise deletion, 1000 bootstrap replicates). The Bayesian phylogenetic tree was generated via the Bayesian inference method using MrBayes software. 

### 2.4. Transformation of Tobacco Leaves

For transformation of tobacco leaves, agrobacterium-mediated transient transformation was used based on a previous method [32]. Agrobacterium strain EHA 105 containing individual constructs was grown on YEP solid medium supplemented with rifampicin (60 μg·mL^−1^) and kanamycin (50 μg·mL^−1^) at 28 °C for 48 h. The selective EHA 105 were then inoculated in 20 mL induction medium containing 1× AB salts (1 g·L^−1^ NH_4_Cl, 0.3 g·L^−1^ MgSO_4_·7H_2_O, 0.15 g·L^−1^ KCl, 0.01 g·L^−1^ CaC1_2_, 0.0025 g·L^−1^ FeSO_4_·7H_2_O), 2 mM phosphate, 1% glucose, 20 mM MES (2-(N-morpholino) ethanesulfonic acid, pH 5.5), 100 μM acetosyringone, rifampicin and kanamycin. After overnight culture at 28 °C, agrobacteria were centrifuged (15 min, 3000× *g*) and resuspended in 10 mM MES (pH 5.5) plus 10 mM MgSO_4_ solution as well as 100 μM acetosyringone, and agrobacterial suspension was adjusted to a final OD_600_ of 0.8 for agroinfiltration. A total of 100 μL of agrobacterial suspension was infiltrated into intercellular spaces of intact tobacco leaves using a 1 mL plastic syringe. After agroinfiltration, tobacco plants were covered with transparent plastic bags and maintained in a growth chamber at 25 °C under a 16 h light/8 h dark cycle for 24–48 h.

### 2.5. Gene Expression Analysis

For tissue-specific expression patterns analysis of *AktWRKY12*, the *A. trifoliata* tissues of bud, young leaves, mature leaves, female flowers, male flowers and young fruit were collected for RNA isolation using the method mentioned above. For infection analysis, three different *A. trifoliata* varieties leaves, including C01 (wile type), I02 (susceptible variety) and H05 (resistance variety), were infected with *C. acutatum* for 6 h and then collected for RNA isolation. For heterologous over-expression analysis, the tobacco leaves were infiltrated with agrobacterium containing 35S::*AktWRKY12* overexpression construct. After growing 36 h in a growth chamber, the tobacco leaves were sprayed with spores of *C. acutatum*. After infection for 6 h, the agroinfiltrated area of tobacco leaves was collected for RNA isolation. In this study, a series of lignin synthesis pathway key enzyme genes and *pathogenesis-related genes* were selected for an expression level test. The expression levels of all detected genes and *AktWRKY12* were based on the qRT-PCR results. qRT-PCR was performed using Mastercycler ep realplex (Eppendorf, Hamburg, Germany) with SYBR Premix Ex TaqTM II (TaKaRa, Dalian, China). *A. trifoliata 18S* and tobacco *Ubiquitin* were used as internal reference, respectively. Each qPCR assay was repeated three times. The expression level of genes was calculated using the 2^−ΔΔCT^ method. Two-tailed Student’s *t*-test (*p* < 0.05) was used to detect the significant difference in relative expression of each gene (Microsoft Excel 2007). Gene-specific primers for quantitative real-time PCR are listed in Table S1.

## 3. Results

### 3.1. cDNA Cloning and Characterization Analysis

**Figure 1:**
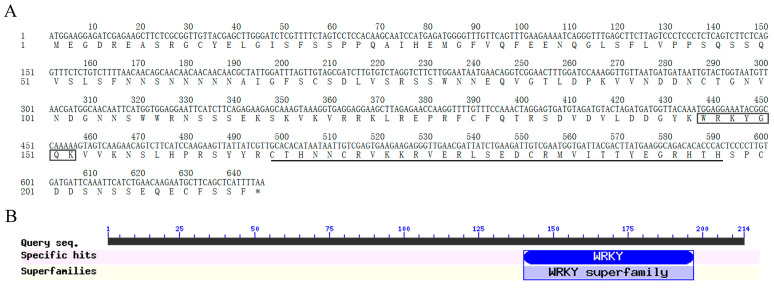
Structure and sequence analysis of AktWRKY12. (**A**) CDS sequence and deduced protein sequence of AktWRKY12. The black box represents conserved heptapeptide WRKYGQK, and the underline represents zinc-finger motif. (**B**) CDD analysis showing the conserved domain of WRKY proteins.

The previous RNA-Seq analysis results showed that the expression level of several WRKY transcriptional factor genes were significantly increased after infection with *C. acutatum* for 6 h in *A. trifoliata* leaves, suggesting that these WRKY gene members might be involved in plant responses to the pathogen attack. In these genes, a *AktWRKY* gene members were significantly induced in all three test *A. trifoliata* varieties leaves after *C. acutatum* infection, especially in the susceptible variety. The ORF of this *AktWRKY* gene member was predicted using TBtools, according to the assembly result of RNA-Seq data [33]. The NCBI blast online tools were used to validate the potential ORF sequences through alignment with other WRKY12s sequences. A pair of special primers was designed according to the ORF prediction results, and PCR amplification of this WRKY gene member was performed. A WRKY-type gene, named *AktWRKY12,* was successfully isolated from *A. trifoliata* leaves, which contained an ORF composed of 645 nucleotides encoding 214 amino acid residues (Figure 1A, Figure S1 and Table S2). The estimated molecular weight of the deduced protein was 24.49 kDa, while the isoelectric point was 7.55. By conducting a search in NCBI-CDD (https://www.ncbi.nlm.nih.gov/cdd, accessed on 10 April 2022), results showed that the predicted protein has a WRKY domain at the C-terminal of the amino acid sequence, followed by a C_2_H_2_ zinc-finger motif (Figure 1B). Subcellular localization prediction analysis using CELLO, an online subcellular localization predictor, suggested that this protein may be present in the nucleus. Gene ontology prediction results indicated that the main molecular function of AktWRKY12 may be DNA binding transcription factor activity, and the biological process of AktWRKY12 was involved in the regulation of gene expression and metabolic processes.

### 3.2. The Structure and Phylogeny of AktWRKY12

**Figure 2:**
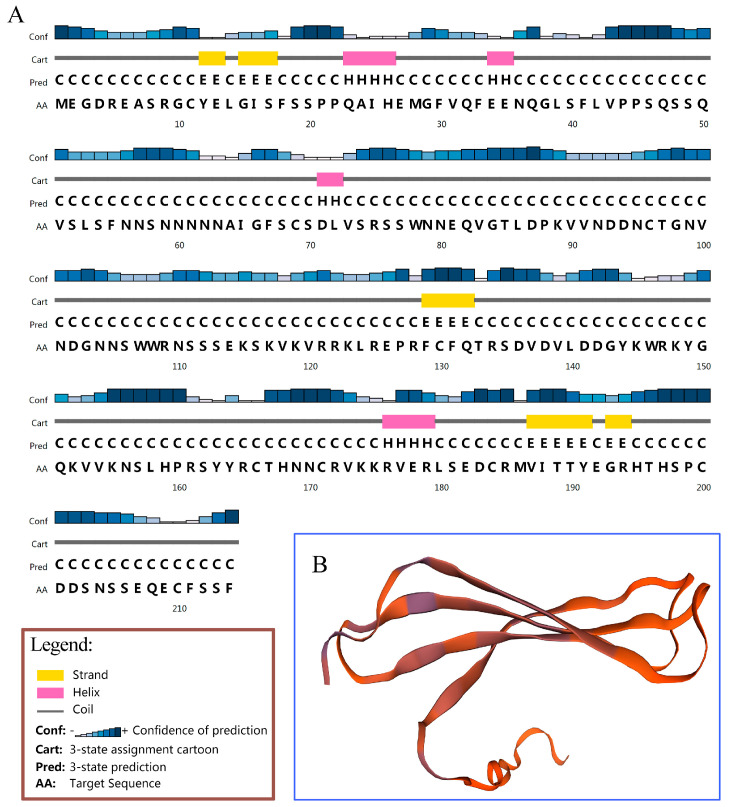
Secondary structure (**A**) and 3D structure (**B**) of the AktWRKY12 protein.

**Figure 3:**
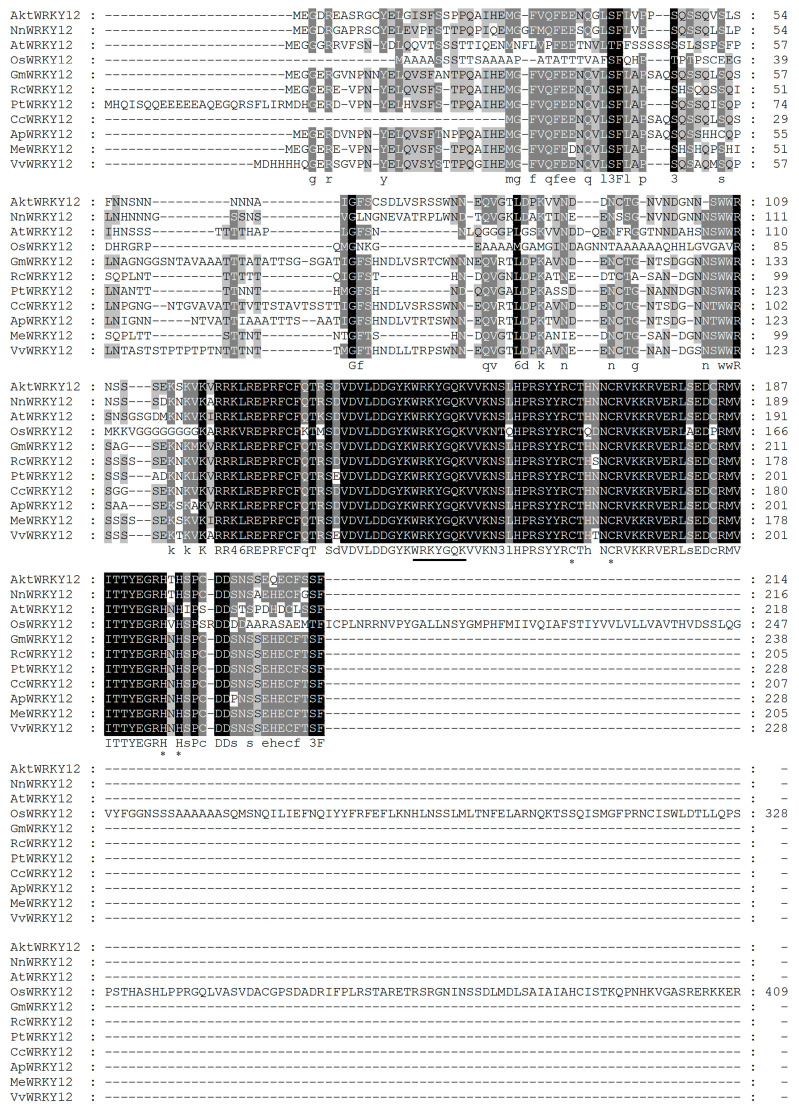
Alignment of AktWRKY12 and other WRKY12s. The sequences used were from *Nelumbo nucifera* WRKY12 (XP_010258736.1), *Arabidopsis thaliana* WRKY12 (OAP07211.1), *Oryza sativa* WRKY12 (AAQ20912.1), *Glycine max* WRKY12 (XP_003521060.1), *Ricinus communis* WRKY12 (XP_015579263.1), *Populus trichocarpa* WRKY12 (XP_006375168.1), *Cajanus cajan* WRKY12 (KYP41601.1), *Abrus precatorius* WRKY12 (XP_027337675.1), *Manihot esculenta* WRKY12 (XP_021623186.1) and *Vitis vinifera* WRKY12 (XP_002270527.1). Underline represents the heptapeptide WRKYGQK. The * indicates the cysteines and histidine of zinc-finger motif.

**Figure 4:**
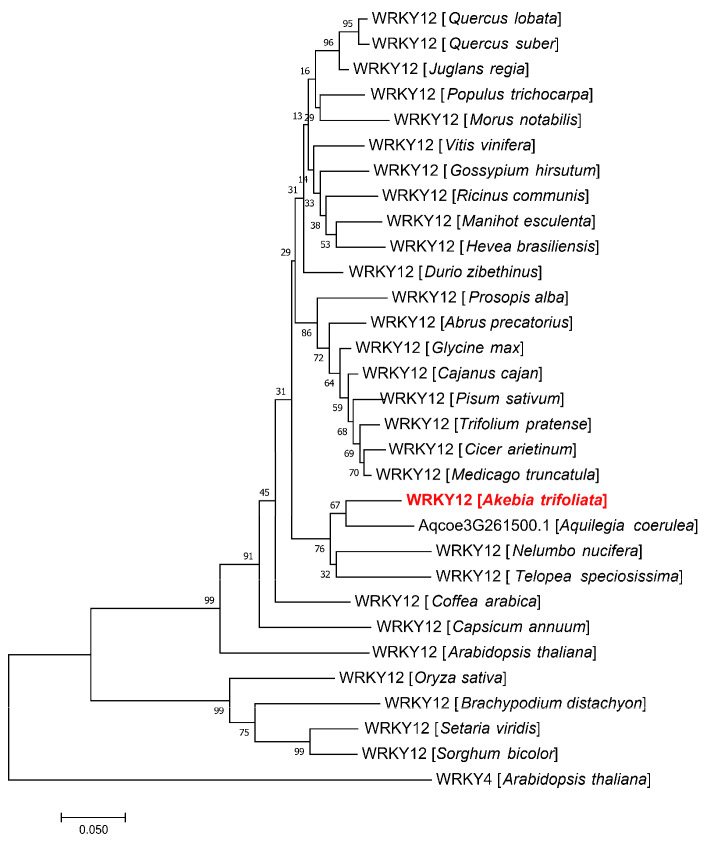
Phylogenetic tree (NJ) constructed via the protein sequence of the AktWRKY12 and other WRKY12 proteins. AtWRKY4 was used as an outgroup.

The secondary structure analysis result showed that the AktWRKY12 protein was composed of four helixes and five β-strands *via* online protein structure prediction (Figure 2A). A 3D molecular model of AktWRKY12 was generated using SWISS MODEL, and results showed that the sequence identity between AktWRKY12 and the template (WRKY4, SMTL id: wj2.1.A) was 62.69%, suggesting that the 3D model of the AktWRKY12 protein was reasonable and belonged to the WRKY family (Figure 2B). As shown in Figure 3, consistent with 10 WRKY12s in other plants, the sequence alignment results showed that AktWRKY12 contained highly conserved WRKY domains followed by a C-X_4_-C-X_23_-H-X-H type zinc finger motif. The alignment of the protein sequence revealed that the WRKY domains in AktWRKY12 and other WRKY12s were strongly conserved, although the N and C terminal sequences of AktWRKY12 showed significant divergence from other WRKY12s. An NJ phylogenetic tree constructed from AktWRKY12 and other WRKY12 proteins was divided into two main clades, monocot and dicot. Obviously, AktWRKY12 belongs to the dicot branch, which was consistent with its phylogenetic classification. Further, results revealed that AktWRKY12 is close to Aqcoe3G261500 (*Aquilegia coerulea*), TsWRKY12 (*Telopea speciosissima*) and NnWRKY12 (*Nelumbo nucifera*), since these four sequences were clustered to a consistent clade (Figure 4 and Figure S2). Moreover, alignment analysis showed the amino acid sequence identity between AktWRKY12 and Aqcoe3G261500 as 79.8%, while the sequence proximity between AktWRKY12 and NnWRKY12 was 76% (Figure 3). Therefore, AktWRKY12 could be classified as a member of group II-c of WRKY transcription factor superfamily, based on the previous classification method [34].

### 3.3. Expression Patterns

**Figure 5:**
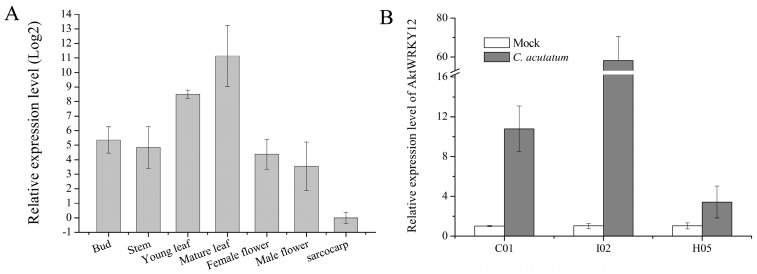
Expression patterns of *AktWRKY12*. (**A**) Expression levels of *AktWRKY12* in different tissues. Bars represent the standard error of the mean. (**B**) Expression pattern of *AktWRKY12* in response to *C. acutatum* inoculation in three *A. trifoliata* varieties. C01, wild type; I02, a susceptible variety; H05, a disease resistance variety.

To investigate the potential physiological functions of *AktWRKY12*, the tissue-specific expression patterns of *AktWRKY12* were performed. Results revealed that the *AktWRKY12* gene can be detected in all tested tissues, including the bud, stem, young leaf, mature leaf, female flower, male flower and sarcocarp. The expression level of *AktWRKY12* was highest in mature leaves of *A. trifoliata*, followed by young leaves, and it was lowest in sarcocarp (Figure 5A). To further investigate the potential functions of the *AktWRKY12* gene in response to pathogen infection, a qRT-PCR analysis was performed to detect the expression level of the *AktWRKY12* gene in three different *A. trifoliata* varieties after *C. acutatum* infection (Figure 5B). Results revealed that the expression levels of *AktWRKY12* significantly increased in all three *A. trifoliata* varieties leaves with pathogen infection, especially in the leaves of the I02 variety (a susceptible variety of *A. trifoliata*).

### 3.4. Heterologous Over-Expression of AktWRKY12 in Tobacco Resulted in Suppressed Expression of Lignin Synthesis Pathway Key Enzyme Genes

**Figure 6:**
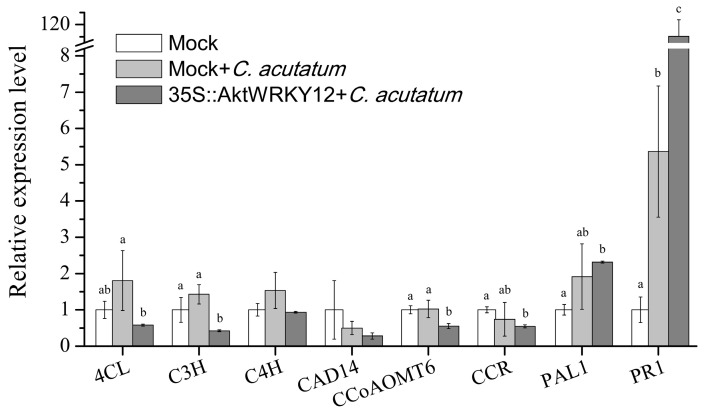
Expression analysis of phenylpropanes metabolism enzyme genes and defense-associated genes in *AktWRKY12*-OE tobacco leaves. Empty construct was used as mock. Ubiquitin was served as a loading control.

To examine whether a change in *AktWRKY12* expression would affect pathogen defense, a 35S::*AktWRKY12* construct was introduced into the tobacco leaf cell *via* Agrobacterium-mediated transient transformation. Compared to the mock (empty construct), the transcript level of some enzyme genes in the phenylpropanes metabolic pathway, including *4CL*, *C3H*, *C4H* and *PAL1*, increased after infection with *C. acutatum* for 6 h (Figure 6). Although the expression levels of *PAL1* (which encode a key enzyme at the first step of the phenylpropanoid path) slightly increased, three lignin synthesis pathway key enzyme genes (*4CL*, *C3H* and *CCoAOMT6*) were significantly suppressed in *AktWRKY12*-OE tobacco leaves compared to that in non-transgenic tobacco after infection with *C. acutatum* for 6 h (*p* < 0.05). Moreover, other test lignin synthesis pathway key enzyme genes (*C4H*, *CAD14* and *CCR*) were also slightly suppressed in *AktWRKY12*-OE tobacco leaves after pathogen treatment. These results demonstrate that AktWRKY12 was involved in pathogens responsive in *A. trifoliata*, and they might play a role in the lignin synthesis pathway, which is consistent with previous work [27]. In addition, the expression of orthologs of lignin synthesis pathway key enzyme genes in *A. trifoliata* were analyzed in different organs and varieties based on RNA-seq results (Figures S3 and S4). Results showed that the transcription levels of most of lignin synthesis pathway key enzyme genes were low in the susceptible variety of *A. trifoliata*, and their expression levels were also down-regulated after *C. acutatum* infection (Figure S4). Interestingly, *PR1*, which was part of the plant’s natural defense response against pathogen attack, was significantly up-regulated 100.9-fold and 5.4-fold in *AKtWRKY12*-OE and non-transgenic tobacco leaves after *C. acutatum* infection compared to control, respectively (*p* < 0.05).

## 4. Discussion

The WRKY TFs comprise one of the largest plant-specific families of transcription factors. The first WRKYs, which was first named SPF1, was isolated from sweet potato (*Ipomoea batatas*). Since then, many WRKY genes have been isolated, identified and functionally characterized in a variety of plants, such as *Arabidopsis thaliana* (74), *Brachypodium distachyon* (86), *Populus* (100) and *Oryza sativa* (109) [15,35,36,37]. Previous studies have revealed that WRKY TFs function as positive or negative regulators during plant disease response. Most WRKY domains can recognize and bind W-box *cis*-acting elements in the promoter region of target genes related to the SA signaling pathway, such as *PR* genes [38]. For example, plants over-expressing *CaWRKY22* and *AtWRKY70* showed constitutive expression of *PR* genes, while over-expression *WRKY4* in Arabidopsis resulted in greatly increased susceptibility of plants to bacterial pathogens and down-regulated pathogen-induced *PR1* gene expression [19,39,40]. On the other hand, WRKY TFs have been found to be involved in regulating the phenylpropanes metabolic pathway, including flavonoids, antitoxin and lignin synthesis [41,42]. Over-expressing *VvWRKY2* in tobacco seedlings exhibited altered expression of lignin biosynthesis-related key enzyme genes, suggesting that VvWRKY2 played a role in the regulation of grape lignification, which might be a response to biotic or abiotic stresses [42]. A study on OsWRKY89 indicated that the over-expression of the *OsWRKY89* gene increased the resistance of rice to the *Magnaporthe oryzae* with an accompanying increase in lignification in culms [43]. Moreover, numbers of *WRKY* TFs have been isolated in some medicinal plants, such as *Artemisia annua* and *Salvia miltiorrhiza* [44,45]. Despite the medical and edible value of *A. trifoliata* being attractive, the yield and quality of the medicinal materials and fruit of *A. trifoliata* was very low because of the disease susceptibility of *A. trifoliata* trees. At the moment, no disease-resistance-related *WRKY* gene has previously been characterized in *A. trifoliata*. In this study, we cloned a fungi-induced *WRKY* gene from *A. trifoliata* (Figure 1). Conserved domain and sequence alignment analysis of the full-length deduced protein clearly exhibited that AktWRKY12 contained a C-terminal WRKY domain followed by a C_2_H_2_ zinc-finger motif and belonged to the group II-C WRKY family (Figure 1, Figure 2 and Figure 3). The WRKY domain sequences of AktWRKY12 and other WRKY12s were almost identical, implying a possible functional similarity among them, and AktWRKY12 might play roles in the phenylpropanes metabolic pathway and disease response [27,46]. Gene expression patterns in different organs and tissues might be due to their physiological functions. To further investigate the potential physiological functions of AktWRKY12, the tissue-specific expression patterns of *AktWRKY12* were investigated (Figure 5A). Results showed that the *AktWRKY12* gene was expressed in all the tissues tested, especially in leaves. Many WRKY transcription factors exhibit greatly uneven distribution among different tissues to exert different physiological functions [47]. For instance, AtWRKY12 played a critical role in pith secondary wall formation, which was abundant in pith and cortex cells of stem and hypocotyls. These results implied that AktWRKY12 might be involved in leaf development.

Previous studies have shown that WRKY12 played significant roles in plant growth and development and abiotic/biotic stress response. For instance, *AtWRKY12*, which was abundant in stem and hypocotyls, has been reported to play a key role in secondary wall formation of pith [27]. Li et al. found that disrupting the expression of AtWRKY12 led to a delayed flowering time, suggesting that WRKY12 mediated the role of GA3 in controlling flowering time to a certain extent [48]. WRKY12 directly targeted GSH1, indirectly inhibited the expression of the PC synthesis-related gene, and negatively regulated the accumulation and tolerance of Cd in Arabidopsis [49]. In *Brassica rapa*, the expression level of *BrWRKY12* increased after pathogen treatment, and over-expression *BrWRKY12* could enhance disease resistance through transcriptional activation of defense-related genes, contrary to the function of WRKY12 in Arabidopsis [46]. Since the expression level of *WRKY12* was significantly induced by *C. acutatum* infection in *A. trifoliata* leaves (Figure 5B), and AktWRKY12 showed high conserved WRKY domains to other WRKY12, we speculated that AktWRKY12 might play a role in regulating plant disease responsive genes expression. Furthermore, the qPCR results showed that three enzyme genes in the lignin synthesis pathway were obviously suppressed in tobacco leaves overexpressing *AktWRKY12* (Figure 6). These results were consistent with a previous report wherein WRKY12 acted as a negative regulator in controlling the biosynthesis of the xylan, cellulose and lignin [27]. Therefore, the expression level of *AktWRKY12* was significantly upregulated in a susceptible variety of *A. trifoliata* after *C. acutatum* infection, which negatively regulated the expression of key enzyme genes in lignin synthesis (Figure 6 and Figure S4). Interestingly, the expression level of *AktWRKY12* in I02 (a susceptible variety of *A. trifoliata*) was higher than other varieties, implying that AktWRKY12 was pathogen inducible and that it might play an important role in response to pathogen attacks. Since lignin synthesis was inhibited in the susceptible variety of *A. trifoliata*, the plant became more vulnerable to pathogens. On the other hand, plants may recruit other disease-resistant pathways to resist further invasion by pathogens, such as an SA-mediated defense response. This might be a possible explanation for why the PR1 gene was up-regulation in *AktWRKY12*-OE tobacco leaves after pathogen treatment.

## 5. Conclusions

A novel WRKY transcription factor designated as *AktWRKY12* was isolated from *A. trifoliata*, an important Chinese traditional medicinal plant. *AktWRKY12* was significantly induced in *A. trifoliata* leaves after pathogen infection. Heterologous over-expression of *AktWRKY12* in tobacco resulted in suppressed expression of key enzyme genes in lignin synthesis. Thus, we primarily conclude that AktWRKY12 might play a negative role in *A. trifoliata* responding to biotic stress by regulating the expression of lignin synthesis key enzyme genes during pathogen infection.

## Data Availability

All data generated or analyzed during this study were included in this published article. The RNA-seq raw data were obtained from Genbank (Accession No. SRR12930913, SRR12930914, SRR12930915, SRR12930911, SRR12930912, SRR12930922, SRR12930919, SRR12930920, SRR12930921, SRR12930939, SRR12930940, SRR12930941, SRR12930916, SRR12930917 and SRR12930918).

## Supplementary Materials

The following supporting information can be downloaded at https://www.mdpi.com/article/10.3390/genes14051015/s1, Figure S1: The gene structure of *AktWRKY12*; Figure S2: Phylogenetic analysis of the deduced amino acid sequence of AktWRKY12 and other WRKY12 proteins; Figure S3: The expression pattern of lignin synthesis pathway key enzyme genes and PR genes of *A. trifoliata* in different tissues; Figure S4: The expression pattern of lignin synthesis pathway key enzyme genes and PR gene in three *A. trifoliata* varieties after *C. acutatum* infection; Table S1: The list of qRT-PCR primers of test genes and Table S2: The CDS and amino sequence of AktWRKY12.
