# Supplementary material for: Molecular Cloning and Characterization of WRKY12, A Pathogen Induced WRKY Transcription Factor from Akebia trifoliata
Source: Genes (Basel). 2023 Apr 29;14(5):1015. doi: 10.3390/genes14051015 (PMC10217843; doi:10.3390/genes14051015)
Supplement: Supplementary file 1 [file genes-14-01015-s001.zip › Figure S3. tissue-specific.pdf]

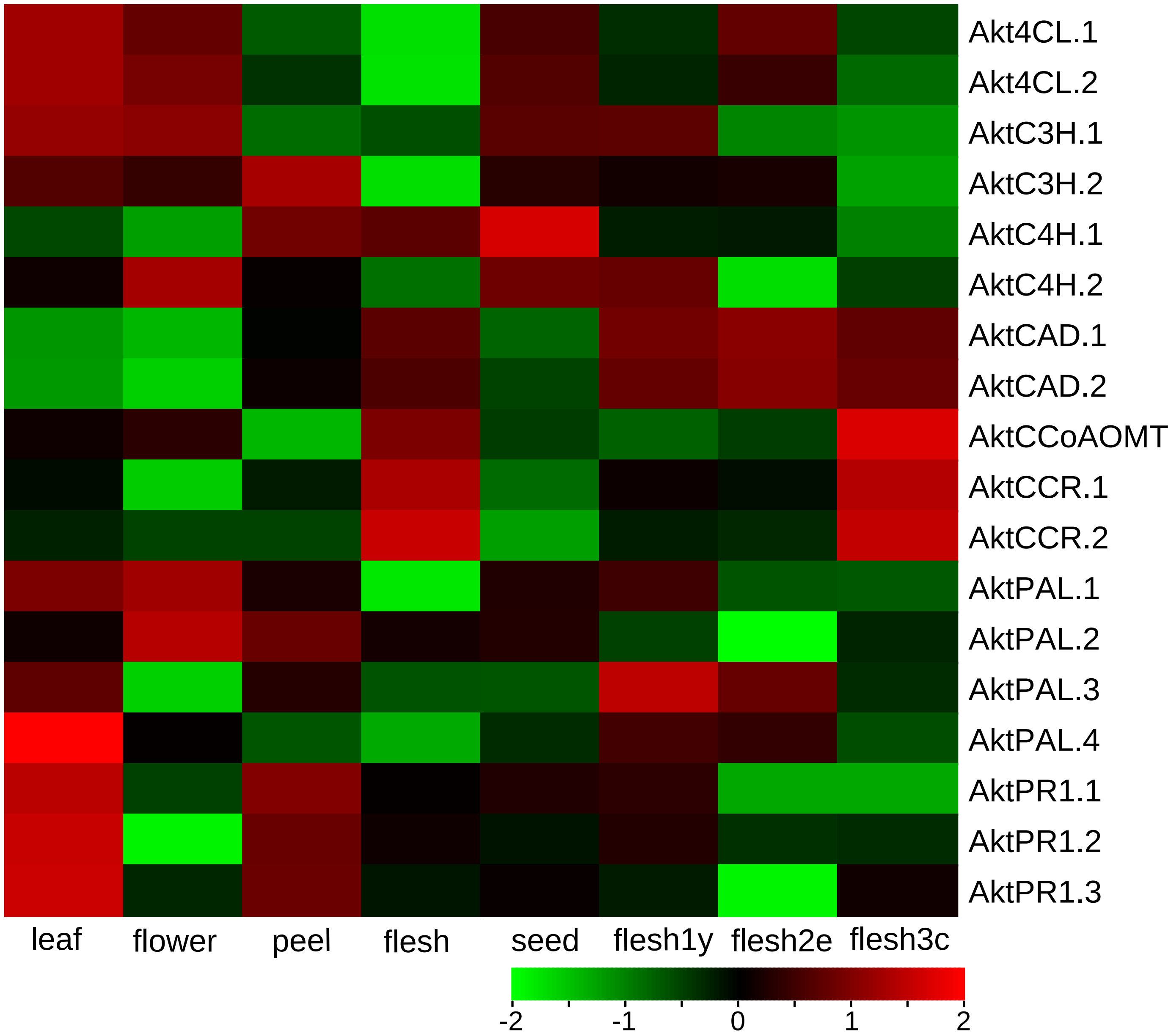

Figure S3 The expression pattern of lignin synthesis pathway key enzyme genes and PR genes of *A. trifoliata* in different tissues. flesh1y, flesh2e and flesh3c represent three different stage of fruit (young, enlargement and coloring, respectively).
