# Supplementary material for: Molecular Cloning and Characterization of WRKY12, A Pathogen Induced WRKY Transcription Factor from Akebia trifoliata
Source: Genes (Basel). 2023 Apr 29;14(5):1015. doi: 10.3390/genes14051015 (PMC10217843; doi:10.3390/genes14051015)
Supplement: Supplementary file 1 [file genes-14-01015-s001.zip › Table S1. primer.pdf]

Table S1. The list of qRT-PCR primers of test genes.

Gene Name	Forward Primer	Reverse Primer
Nt4CL	AGGGGCATGTGGTACTGTTG	CGCCAGTGTGTAACCATCCT
NtC3H	CCAACAAAGTGGAGGAGCCA	TCTTCTTGGGCCTTCTGCTG
NtC4H	AGAGGAGAAGCACGTTGAGG	ATTGCCCGCCTTTCTCTGTT
NtCAD14	GGCTGCTAGAGACCCTTCTG	TCTGGTCCTACCTCCACCAC
NtCCoAOMT6	CGTAATCCAAAAGGCTGGCG	AGCCAATCACACCACCAACT
NtCCR	ATGTGATAACGGCAGCAGCA	CTCCTTTCTCCCTTGCCTCG
NtMAPK3	ACATCCTCGCCAGCAGTTAG	GGAGAATGGAACAGGGCAGA
NtMAPK6	ATACAGTCGGAGGACCAGCA	GGGTGGCTCAAAAGGTCCAA
NtPAL1	ACAATCAGGGACAAGGGCAG	GCCGACCTGTGAGTAAACCA
NtPR1	TTCTCATGGTCAATACGGCGA	ACATCCAACACGAACCGAGT
NtUbiquitin	CAGGGAAGACCATAACCCTAGA	CTCGAAGCCTCAGCACAAGA
AktWRKY12	GTCCCTCCCTCTCAGTCTTC	CACCATGAATTGTTGCCATC
Akt18S	CGGCTACCACATCCAAGGAA	TGTCACTACCTCCCCGTGTCA
