# Supplementary material for: Molecular Cloning and Characterization of WRKY12, A Pathogen Induced WRKY Transcription Factor from Akebia trifoliata
Source: Genes (Basel). 2023 Apr 29;14(5):1015. doi: 10.3390/genes14051015 (PMC10217843; doi:10.3390/genes14051015)
Supplement: Supplementary file 1 [file genes-14-01015-s001.zip › Table S2. AktWRKY12 seq.pdf]

Table S2. The coding sequence and protein sequence of AktWRKY12 in different varieties.

Variety	Coding sequence	Protein sequence
**H05**	ATGGAAGGAGATCGAGAAGCTTCTCGCGGTTGTTACGAGCTTGGGATCTCGTTTTCTAGTCCTCCACAAGCAATC<br>CATGAGATGGGGTTTGTTCAGTTTGAAGAAAATCAGGGTTTGAGCTTCTTAGTCCCTCCCTCTCAGTCTTCTCAGG<br>TTTCTCTGTCTTTTAACAACAGCAACAACAACAACAACGCTATTGGATTTAGTTGTAGCGATCTTGTGTCTAGGTC<br>TTCTTGGAATAATGAACAGGTCGGAACTTTGGATCCAAAGGTTGTTAATGATGATAATTGTACTGGTAATGTTAAC<br>GATGGCAACAATTCATGGTGGAGGAATTCATCTTCAGAGAAGAGCAAAGTAAAGGTGAGGAGGAAGCTTAGAGA<br>ACCAAGGTTTTGTTTCCAAACTAGGAGTGATGTAGATGTACTAGATGATGGTTACAAATGGAGGAAATACGGCCA<br>AAAAGTAGTCAAGAACAGTCTTCATCCAAGAAGTTATTATCGTTGCACACATAATAATTGTCGAGTGAAGAAGAG<br>GGTTGAACGATTATCTGAAGATTGTCGAATGGTGATTACGACTTATGAAGGCAGACACACCCACTCCCCTTGTGAT<br>GATTCAAATTCATCTGAACAAGAATGCTTCAGCTCATTTTAA	MEGDREASRGCYELGISFSSPPQAIHEM<br>GFVQFEENQGLSFLVPPSQSSQVSLSFNN<br>SNNNNNAIGFSCSDLVSRSSWNNEQVGT<br>LDPKVVNDDNCTGNVNDGNNSWWRNS<br>SSEKSKVKVRRKLREPRFCFQTRSDVDV<br>LDDGYKWRKYGQKVVKNSLHPRSYYR<br>CTHNNCRVKKRVERLSEDCRMVITTYE<br>GRHTHSPCDDSNSSEQECFSSF*
**C01**	ATGGAAGGAGATCGAGAAGCTTCTCGCGGTTGTTACGAGCTTGGGATCTCGTTTTCTAGTCCTCCACAAGCAATC<br>CATGAGATGGGGTATGTTCAGTTTGAAGAAAATCAGGGTTTGAGCTTCTTAGTCCCTCCCTCTCAGTCTTCTCAGG<br>TTTCTCTGTCTTTTAACAACAGCAACAACAACAACAACGCTATTGGATTTAGTTGTAGCGATCTTGTGTCTAGGTC<br>TTCTTGGAATAATGAACAGGTCGGAACTTTGGATCCAAAGGTTGTTAATGATGATAATTGTACTGGTAATGTTAAC<br>GATGGCAACAATTCATGGTGGAGGAATTCATCTTCAGAGAAGAGCAAAGTAAAGGTGAGGAGGAAGCTTAGAGA<br>ACCAAGGTTTTGTTTCCAAACTAGGAGTGATGTAGATGTACTAGATGATGGTTACAAATGGAGGAAATACGGCCA<br>AAAAGTAGTCAAGAACAGTCTTCATCCAAGAAGTTATTATCGTTGCACACATAATAATTGTCGAGTGAAGAAGAG<br>GGTTGAACGATTATCTGAAGATTGTCGAATGGTGATAACGACTTATGAAGGCAGACACACCCACTCCCCTTGTGAT<br>GATTCAAATTCATCTGAACAAGAATGCTTCAGCTCATTTTAA	MEGDREASRGCYELGISFSSPPQAIHEM<br>GYVQFEENQGLSFLVPPSQSSQVSLSFNN<br>SNNNNNAIGFSCSDLVSRSSWNNEQVGT<br>LDPKVVNDDNCTGNVNDGNNSWWRNS<br>SSEKSKVKVRRKLREPRFCFQTRSDVDV<br>LDDGYKWRKYGQKVVKNSLHPRSYYR<br>CTHNNCRVKKRVERLSEDCRMVITTYE<br>GRHTHSPCDDSNSSEQECFSSF*
**I02**	ATGGAAGGAGATCGAGAAGCTTCTCGCGGTTGTTACGAGCTTGGGATCTCGTTTTCTAGTCCTCCACAAGCAATC<br>CATGAGATGGGGTATGTTCAGTTTGAAGAAAATCAGGGTTTGAGCTTCTTAGTCCCTCCCTCTCAGTCTTCTCAGG<br>TTTCTCTGTCTTTTAACAACAGCAACAACAACAACAACGCTATTGGATTTAGTTGTAGCGATCTTGTGTCTAGGTC<br>TTCTTGGAATAATGAACAGGTCGGAACTTTGGATCCAAAGGTTGTTAATGATGATAATTGTACTGGTAATGTTAAC<br>GATGGCAACAATTCATGGTGGAGGAATTCATCTTCAGAGAAGAGCAAAGTAAAGGTGAGGAGGAAGCTTAGAGA<br>ACCAAGGTTTTGTTTCCAAACTAGGAGTGATGTAGATGTACTAGATGATGGTTACAAATGGAGGAAATACGGCCA<br>AAAAGTAGTCAAGAACAGTCTTCATCCAAGAAGTTATTATCGTTGCACACATAATAATTGTCGAGTGAAGAAGAG<br>GGTTGAACGATTATCTGAAGATTGTCGAATGGTGATAACGACTTATGAAGGCAGACACACCCACTCCCCTTGTGAT<br>GATTCAAATTCATCTGAACAAGAATGCTTCAGCTCATTTTAA	MEGDREASRGCYELGISFSSPPQAIHEM<br>GYVQFEENQGLSFLVPPSQSSQVSLSFNN<br>SNNNNNAIGFSCSDLVSRSSWNNEQVGT<br>LDPKVVNDDNCTGNVNDGNNSWWRNS<br>SSEKSKVKVRRKLREPRFCFQTRSDVDV<br>LDDGYKWRKYGQKVVKNSLHPRSYYR<br>CTHNNCRVKKRVERLSEDCRMVITTYE<br>GRHTHSPCDDSNSSEQECFSSF*
