# Supplementary figures and images for: Molecular Cloning and Characterization of WRKY12, A Pathogen Induced WRKY Transcription Factor from Akebia trifoliata
Source: Genes (Basel). 2023 Apr 29;14(5):1015. doi: 10.3390/genes14051015 (PMC10217843; doi:10.3390/genes14051015)

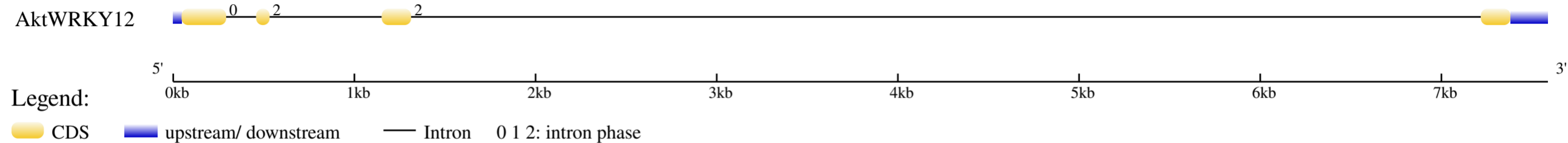

Figure S1 The gene structure of AktWRKY12.

Supplement: Supplementary file 1 [file genes-14-01015-s001.zip › Figure S1. gene structure.pdf]

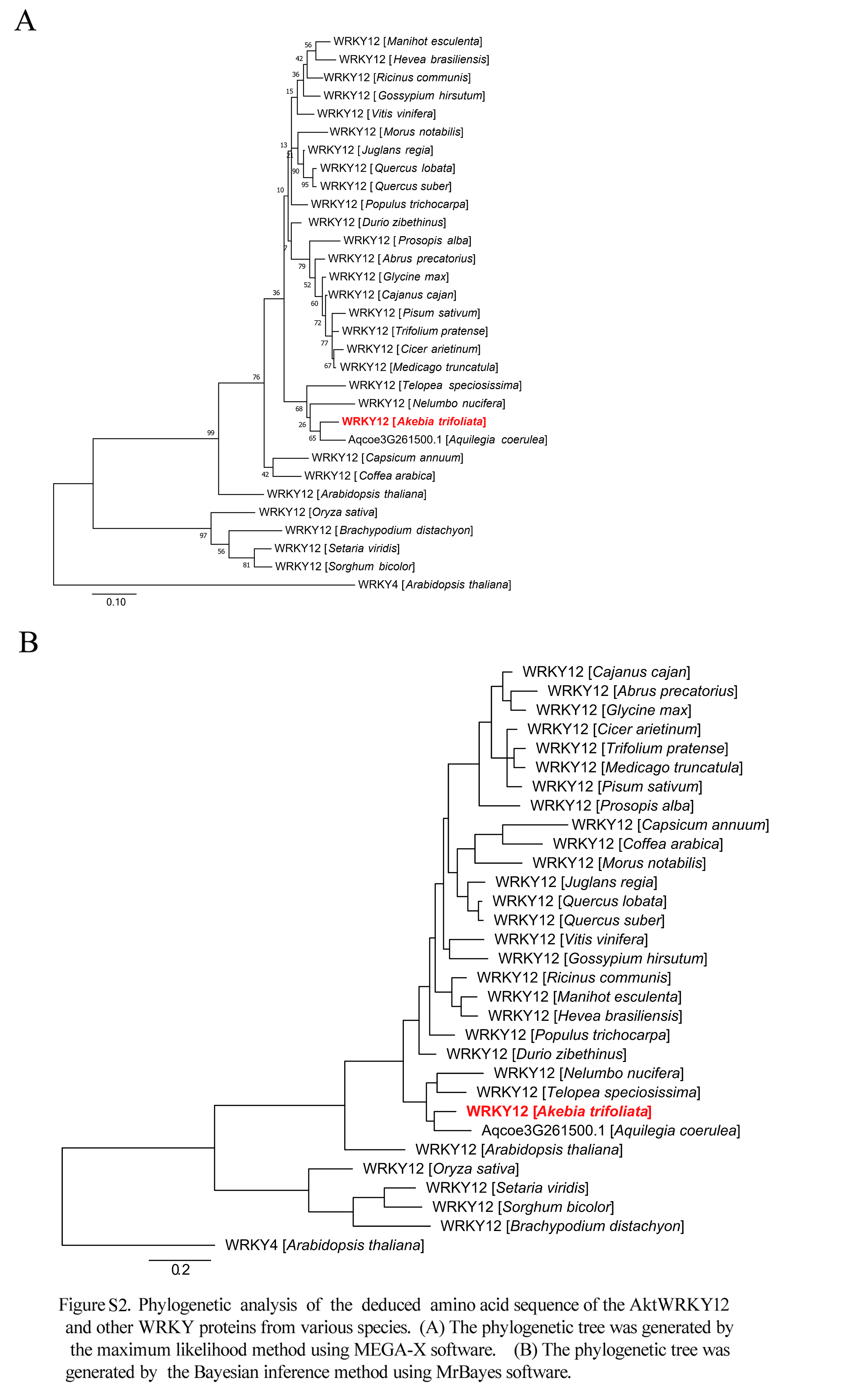

Supplement: Supplementary file 1 [file genes-14-01015-s001.zip › Figure S2. Phylogenetic tree.tif]
